# Visual Outcome of Descemet Membrane Endothelial Keratoplasty during the Learning Curve in Initial Fifty Cases

**DOI:** 10.1155/2019/5921846

**Published:** 2019-03-17

**Authors:** Sanjay K. Singh, Sanjeeta Sitaula

**Affiliations:** ^1^Department of Cornea Clinic, Biratnagar Eye Hospital, Biratnagar 56613, Nepal; ^2^B.P.Koirala Lions Centre for Ophthalmic Studies, Maharajgunj Medical Campus, Institute of Medicine, Kathmandu 44600, Nepal

## Abstract

This study was performed to evaluate the clinical outcomes of the first fifty patients who underwent Descemet membrane endothelial keratoplasty (DMEK) during the 3-month postoperative period and to describe the challenges encountered during the learning curve. In this retrospective study, we reviewed the charts of patients who underwent DMEK. All information regarding patient demographics, indication for surgery, preoperative and postoperative visual acuity at 3 months, donor age, and complications encountered intraoperatively and postoperatively was recorded. Donor endothelial cell count at the time of surgery and during the 3-month follow-up was noted. Data were analyzed using SPSS version 17. Fifty eyes of 49 patients were included in the study with majority being female patients (male : female = 2 : 3). Mean age of patients was 56.8 ± 11.4 years with the age range of 22–78 years. The common indications for DMEK were pseudophakic bullous keratopathy –57.1%, Fuchs endothelial dystrophy-34.7%, failed grafts-6.1% (Descemet stripping endothelial keratoplasty (DSEK) and failed penetrating keratoplasty), and others. Preoperative best spectacle-corrected visual acuity was <20/400 in 88% cases. Postoperative best spectacle-corrected visual acuity at 3 months was >20/63 in 41.8% of the cases, and 93% had visual acuity of 20/200 or better. Donor size was 8 mm, and average donor endothelial cell count (ECC) was 2919 ± 253 cells/mm^2^. Average ECC at 3 months postoperatively was 1750 ± 664 cells/mm^2^, which showed a 40% decrease in ECC. The most common encountered complication was graft detachment, which occurred in 16% cases for which rebubbling was done. Regular follow-up and timely identification of graft detachment may prevent the need for retransplantation.

## 1. Introduction

The concept of Descemet membrane endothelial keratoplasty (DMEK) was introduced by Melles in 2002 [1], and the first successful case of DMEK was reported in 2006 for Fuchs endothelial dystrophy by Melles et al. [2, 3]. Since then, DMEK has gained popularity as a surgical option for corneal endothelial disease. The benefits of DMEK over other types of keratoplasty have previously been discussed and include preservation of ocular integrity, earlier visual rehabilitation, and better visual outcome without suture-related ocular surface complications [3–10]. Other advantages of DMEK include reduced risk of graft rejection and cheaper equipment and setup [11–13]. In addition, the donor cornea can be effectively utilized for two lamellar surgeries: deep anterior lamellar keratoplasty (DALK) and DMEK in areas where there is still a scarcity of donor corneas [14]. Outcomes of DMEK are superior compared to Descemet stripping endothelial keratoplasty (DSEK) in terms of providing better visual acuity, more predictable postoperative refractive outcomes, and reduced rate of immune reactions [12, 15, 16]. However, the learning curve is quite steep and is a major hindrance for cornea surgeons to transition from penetrating keratoplasty (PK) or DSEK to DMEK [7, 17]. The major challenges in DMEK involve handling the thin tissue during donor preparation while avoiding tears of the graft, minimizing the loss of endothelial cells during preparation, and intraoperatively unrolling the graft in the proper orientation within the anterior chamber [4, 18, 19]. The most common postoperative complications following DMEK surgery is graft detachment which can be managed by rebubbling [7, 20, 21]. This study was conducted with the aim to describe the clinical outcome of DMEK cases at 3 months performed by a single surgeon and to describe the difficulties and complications encountered during the initial learning curve.

## 2. Materials and Methods

In this observational retrospective single surgeon case series, we included the first 50 eyes of 49 patients that underwent DMEK at Biratnagar Eye Hospital (BEH) from August 2016 to January 2018 who had at least 3 months of follow-up. The surgeon had undergone two 2-day wet lab courses and later practiced the surgical technique using an artificial anterior chamber before performing the surgery in human eyes. Ethical approval for the study was obtained from the Hospital Review Board of Biratnagar Eye Hospital, and this study adheres to the tenets of the Declaration of Helsinki.

All the relevant patient information including age, sex, indication for surgery, surgical procedure, slitlamp findings, intraocular pressure, complications encountered intraoperatively and postoperatively, preoperative and postoperative visual acuity, donor endothelial cell count (ECC), and donor age was recorded. Donor ECC was measured by the Nepal eye bank. The postoperative ECC was measured with a noncontact specular microscope (Nidek CEM-530). Patients with large iris defects, aphakia, and history of pars plana vitrectomy and those who were not followed up through 3 months were excluded from this study. The eyes were operated under peribulbar block followed by ocular massage. The surgical technique used is briefly described here. A backup cornea was always available during graft preparation.

### 2.1. Graft Preparation

Donor corneas with suitable endothelial cell count (ECC) processed from the Nepal Eye Bank and stored in Cornisol corneal storage media (Aurolab, Madurai, India) were used for preparation of the graft by the operating surgeon just before surgery. SCUBA (“submerged cornea, using backgrounds away”) technique which was described first in 2009 [6] was performed under ringer lactate (RL) solution. The donor cornea was placed endothelial side up in a Barron vacuum donor cornea punch 9.5 mm (BPI, USA) and lightly tapped to punch superficially up to the level of Descemet membrane. The donor tissue was then transferred into the Teflon block. The endothelium was scored using a Dr Fogla DMEK scorer (Joja Surgical Private Limited, India) to separate the Descemet over the punched mark by gently rotating the donor cornea over the Teflon block. Using suture tying forceps, around 60% of the Descemet membrane and the endothelium were gently peeled away from the stroma. A 2 mm punch was used to punch the stroma at the site where the endothelium was peeled away. At this point, trypan blue dye (Contacare Ophthalmics and Diagnostics, India) 0.06% was applied over the graft for 10–15 seconds. The excess dye was washed with RL, and the graft was repositioned back over the stroma. The donor cornea was then placed epithelial side up, and the punched corneal cap was removed. The S-mark was placed with S-marker over the Descemet, and the cap was repositioned back. The donor cornea was positioned over 8 mm Barron vacuum punch endothelial side up and punched. The rest of the attached graft was peeled after which the graft spontaneously formed a scroll with endothelium on the outside. The graft was stained with trypan blue for 5 minutes and placed in a glass bowl containing RL. The graft was aspirated in a curved glass pipette (DMEK disposable surgical set, D.O.R.C, the Netherlands) attached to a 3 ml syringe.

### 2.2. Recipient Preparation

Glycerine was placed over the cornea, and the epithelium was debrided whenever necessary for better visualization in the event of an edematous cornea. An 8 mm circular mark was placed over the cornea with an 8 mm trephine marked with dye to delineate the area for Descemetorhexis. A 2.8 mm scleral tunnel incision was made at 12 o'clock, and 3 side ports were created at 3, 6, and 9 o'clock. Descemetorhexis was done with a reverse Sinskey hook (Joja Surgical Private Limited, India) and reverse Rhexis forceps (Joja Surgical Private Limited, India) under cohesive viscoelastic. In cases where the cataract was significant, phacoemulsification was performed and foldable intraocular lens was implanted. An inferior peripheral iridotomy was made with the vitrector. Viscoelastic was completely washed from the anterior chamber prior to insertion of the graft.

The graft was injected into the anterior chamber through the superior scleral incision, and a suture was applied. The graft was unfolded by “Dirisamer technique” [22]. In this technique, two cannulas are used to unfold a single DMEK roll by gently tapping over the outer corneal surface to separate the outer curl of the roll. Once the outer curl unrolls, it was fixated by gentle pressure of one cannula onto the outer corneal surface. Another cannula was used to apply gentle strokes parallel to the roll, to unroll the graft like a carpet without ever directly touching the graft. The orientation of graft was confirmed by observing the S-mark and by observing a positive Moutsouris sign. Once the graft orientation and position was satisfactory, air was injected into AC. The patient was taken to the recovery room and made to lie in a supine position.

The patient was examined after 3 hours to check for pupillary block. If the pupillary block was observed, air was released through the side port under the slit lamp. Postoperatively, each patient was started with a topical steroid antibiotic combination, which was gradually tapered over 2 months and kept at a once-daily dosage thereafter.

Patients were examined preoperatively, on the first postoperative day, at 1 week, 1 month, and at 3 months. At each visit, the best-corrected visual acuity was recorded, and the status of graft attachment or any other complications was noted. ECC and CCT were recorded at 3 months.

Statistical analysis was done using SPSS version 17 statistical software (SPSS Inc, Chicago, Illinois); *P* value <0.05 was considered statistically significant. Association between different variables was tested using Pearsonʼs chi-square test.

## 3. Results

Fifty eyes of 49 patients were included in the study.

### 3.1. Demographic Pattern

There were 20 (40.8%) male and 29 (59.2%) female patients undergoing DMEK surgery. Mean age of the patients undergoing DMEK surgery was 56.82 ± 11.40 years with the age ranging from 22–78 years. Most of the patients (42.85%) were 61–70 years. The most common indication for surgery (Table 1) was pseudophakic bullous keratopathy (57.1%) followed by Fuchs endothelial dystrophy (34.7%). Three patients underwent DMEK for a failed graft: one for failed penetrating keratoplasty and 2 for failed DSEK. One patient who underwent DMEK had iridocorneal endothelial (ICE) syndrome.

Thirty-nine eyes (78%) underwent DMEK alone, whereas 11 eyes (22%) underwent DMEK along with phacoemulsification and foldable intraocular lens implantation at the same sitting.

Mean donor age was 59.8 ± 13.68 years with a range of 33–75 years.

### 3.2. Visual Outcome

Preoperative best spectacle-corrected visual acuity was <20/200 in all cases with 88% cases having visual acuity of <20/400 (Table 2). At third postoperative month, 93% had best spectacle-corrected visual acuity better than 20/200 and 41.8% had better than 20/63 after excluding the 7 eyes that had failed graft (Table 3).

### 3.3. Donor Preparation and Endothelial Cell Count (ECC)

The most common complication while preparing the graft was tearing the edge of the graft while peeling it off the stroma, which occurred in 2 cases. In such situations, the donor cornea was rotated and tearing was initiated from another side. None of the grafts had to be discarded. In one case, the tear was small and was not included by the 8 mm punch. In the other, although the tear extended to the graft, it was small so the graft was still used for DMEK with good visual outcome. The mean donor ECC was 2919 (±253) cells/mm^2^ (range: 2427–3509 cells/mm^2^). Postoperatively, ECC could not be taken in 7 grafts which failed and in 2 grafts where central subepithelial and stromal scarring was present. Five other cases did not have ECC recorded. Among the rest 36 eyes (72%) that had ECC records, the mean postoperative ECC was 1750 (±664) with a range of 689–2757 cells/mm^2^. The mean rate of endothelial cell loss postoperatively was 40.01% compared to preoperative values.

### 3.4. Complications

The list of complications is summarized in Table 4. The most common complication encountered was graft detachment noted in 8 eyes (16%), 3 were identified within 7 days and 5 cases after 7 days. Among these 8 cases, two of the cases missed the 1-week follow-up and returned at postoperative month one with graft detachment. Air injection (rebubbling) was done in all the cases. Rebubbling was not successful for graft reattachment in the 2 eyes (4%) with late presentation. In 4 other cases, there was graft failure despite good graft attachment, and the cornea did not clear at all.

In one case, the graft was oriented upside-down (endothelial side towards the stroma) which failed and repeat DSEK was done. Repeat corneal grafting was done in a total of 6 cases (12%), 1 PK, 2 DMEK, and 3 DSEK. In another case with failed DMEK, repeat surgery was planned, but the patient failed to follow-up.

Pupillary block occurred in 4 (8%) patients noted around 3-4 hours after surgery for which air was released under the slit lamp. Removal of exudates over the pupillary area was done in the first postoperative day for 1 patient (2%).

Persistent epithelial defect was noted in 3 eyes (6%), which was managed by applying bandage contact lens and increasing the frequency of topical lubricating drops. There was one case that developed cystoid macular edema. One patient had developed features of graft rejection at 3 months when he stopped using topical steroids on his own; however, upon restarting steroids, the corneal edema cleared, and the patient gained best-corrected visual acuity of 20/32.

### 3.5. Correlation between Different Variables with Postoperative Visual Acuity

Using the Pearson chi-square test, there was no significant difference in postoperative best spectacle-corrected visual acuity at 3 months between DMEK alone and DMEK combined with phacoemulsification (Table 5). Postoperative visual acuity was found to be significantly different between donor age <50 versus >50 years (Table 6). No significant difference in postoperative best spectacle-corrected visual acuity at 3 months was noted between the various indications for surgery as shown in Table 7.

## 4. Discussion

The literature has pointed out the advantages and superiority of DMEK over PK and DSEK for corneal endothelial pathology [3, 6, 10, 15–17]. Many corneal surgeons now prefer DMEK for diseases of the corneal endothelium [15, 23], but because the technique for graft preparation and graft unfolding within the AC requires a new set of surgical skills, adoption of DMEK surgery comes with learning difficulties. This study was performed to evaluate the clinical and visual outcomes of the initial 50 DMEK cases of a single surgeon and to describe the common difficulties and complications encountered during the learning curve when adopting DMEK. To the best of our knowledge, this is the first report of DMEK from Nepal.

The surgical technique used in our study was as described by Rodríguez‐Calvo‐de‐Mora et al. [9] with some minor modifications such as doing the Descemetorhexis under cohesive viscoelastic, using the S-stamp for graft orientation and loading the graft into the glass injector as a single roll. The graft unfolding technique mostly used was technique 2 (Dirisamer technique) [22], where the single roll graft was unfolded in AC using two cannulas. The major indication for surgery was pseudophakic bullous keratopathy (57%) followed by Fuchs endothelial corneal dystrophy (34.7%), in contrast to other studies where the major indication for DMEK surgery is FECD [9, 24]. BEH is a tertiary eye care center located in the southeast region of Nepal, close to the Indian border where patients from India are allowed to cross freely. BEH serves as a primary referral center for patients with corneal problems from the eastern region of Nepal and from neighboring Indian states. Manual small incision cataract surgery (M-SICS) is a commonly performed procedure as it is cost-effective and has excellent visual outcome [25–27]. However, most patients in this region present with mature cataracts and probably a missed preoperative diagnosis of endothelial disease [28], which may be the cause for frequent occurrence of postoperative Descemet's membrane detachment, corneal edema, and pseudophakic bullous keratopathy, which were the major indications for DMEK in our study.

The preoperative visual acuity was <20/400 in 88% cases, and 100% cases had less than 20/200 as opposed to >20/40 in 38% cases in a study by Rodríguez-Calvo-de-Mora et al. [9]. Most cases presented very late with long-standing stromal and epithelial edema leading to some degree of subepithelial and stromal scarring, resulting in suboptimal postoperative visual acuity despite good graft centration and attachment. In our study, 93% patients obtained postoperative visual acuity better than 20/200 and 41.8% better than 20/63. This postoperative visual acuity was poorer compared to other studies [9, 15, 24]. The factor responsible for this was that we did not exclude preexisting corneal scars due to long-standing corneal edema, which was present in 75% cases. We also included more cases with low preoperative visual acuity, pseudophakic bullous keratopathy, and older patients, which are shown to have poorer visual outcome in other studies [9, 24]. The follow-up duration was only 3 months, so complete visual recovery may be further possible, which was a limitation in our study.

Donor ECC above 2400 was used for DMEK with mean ECC of 2919 cells/mm^2^. The mean postoperative ECC was 1750 cells/mm^2^. The decline in the ECC during the first 3 months was similar to other studies involving DSEK/DSAEK [9, 12, 29]. Our rate of ECC loss was higher than that described by Chaurasiya et al. where they reported a decline by only 26% at 3 months [24]. However, a notable finding was increase in ECC noted in a few patients from 6 weeks to 3 months, and sometimes thereafter, which might be due to endothelial migration and redistribution or simply due to accurate calculation once the corneal edema had cleared at subsequent visits.

The most common complication was graft detachment noted in 16% cases, which was similar to other studies which report a mean rebubble rate of 28.8% (range, 2.4% to 82%) [15]. We did not have anterior segment optical coherence tomography at the time, so in the early postoperative period in the presence of corneal edema, partial graft detachments may have been missed. The late detection of graft detachment may have led to nonclearing of corneal edema in 4 cases where despite graft adherence to the recipient after rebubbling, the cornea failed to clear. The graft was oriented upside-down in 1 case leading to primary graft failure for which repeat DSEK was done. This complication occurred in the initial few cases where due to the haziness of cornea and the poor contrast against the patient's dark brown iris, the graft orientation could not be visualized properly despite the S-marking. One study highlighted the difficulties encountered while doing DMEK in Asian eyes due to the narrow palpebral fissure, small deep set eyes, relatively shallow anterior chamber, and dark iris [30]. The S-stamp has proved to be useful to prevent upside-down graft insertion without an increased risk of endothelial cell loss [31]. Another technique using endoilluminator for identifying graft orientation and enhancing 3-dimensional depth perception within the anterior chamber is helpful in cases with an edematous cornea where light reflexes from graft folds and edges are visualized better [32]. The high rate of pupillary block in our series was due to near-total air fill left in AC postoperatively due to the high rate of graft detachment noted in the initial few cases. One case had postoperative cystoid macular edema, but it could not be determined whether it occurred after DMEK or following complicated cataract with Descemet membrane detachment for which DMEK was done.

No significant difference in visual outcome was noted when we compared the DMEK alone to DMEK combined with phacoemulsification (triple-DMEK), which was similar to findings from another study [24]. However, recently a study has reported that triple-DMEK may be an independent risk factor for postoperative graft detachment [33].

Postoperative visual acuity was found to be significantly better in patients receiving tissue from a donor age >50 compared to tissue from donors <50 years. Previously, it was reported that increased surgical manipulations and longer unfolding times were associated with younger donor grafts and led to more endothelial cell trauma and ECC loss [34]. This finding was supported by another study which reported that younger donor age might be associated with a 3% increase in the risk of a detachment [35]. However, one retrospective study analyzed the records of 1084 cases, where 17% had young donors (<55 years). This study concluded that younger donor age did not affect the clinical outcome negatively within the first postoperative year [19].

We tried to compare the visual outcome among various indications for DMEK surgery; but due to small sample size, the association could not be observed. Previous studies have reported better visual outcome after DMEK in patients with Fuchs endothelial dystrophy than with pseudophakic bullous keratopathy [10].

## 5. Conclusion

DMEK is a useful technique in resource limited setting as the cost of the equipment required is cheaper compared to other lamellar surgeries. Also, the requirement of postoperative steroid is for a shorter duration, which is an important factor in patients with low compliance in a developing country like Nepal and India. There are four major challenges associated with DMEK surgeries: DMEK donor preparation, insertion, unfolding, and early postoperative complications management. After a short-term wet lab course and thorough wet lab practices before starting surgeries on human being, the learning curve is reasonably smooth with a less complication rate.

## Figures and Tables

**Table 1:** Indications for Descemet membrane endothelial keratoplasty (DMEK).

Diagnosis	Frequency	Percent
Fuchs endothelial corneal dystrophy	17	34.7
Pseudophakic bullous keratopathy	28	57.1
Failed grafts (failed PK^*∗*^/DSEK^*∗∗*^)	3	6.1
Others	1	2.0
Total patients	49	100

^*∗*^PK: penetrating keratoplasty. ^*∗∗*^DSEK: Descemet stripping endothelial keratoplasty.

**Table 2:** Preoperative best spectacle-corrected visual acuity among patients undergoing Descemet membrane endothelial keratoplasty.

Preoperative visual acuity	Frequency	Percent
<20/200–20/400	6	12
<20/400-PL^*∗*^	44	88
Total	50	100

^*∗*^PL: perception of light.

**Table 3:** Postoperative visual acuity among patients undergoing Descemet membrane endothelial keratoplasty at 3 months.

Postoperative visual acuity at 3 months	Frequency	Percent
20/20–20/63	18	41.8
<20/63–20/200	22	51.1
<20/200–20/400	2	4.6
<20/400-PL^*∗*^	1	2.3
Total	43	100

^*∗*^PL: perception of light.

**Table 4:** List of complications encountered following Descemet membrane endothelial keratoplasty surgery.

Complications	No. of eyes (%)
Graft detachment	8 (16%)
Graft failure	7 (14%)
Upside-down graft	1 (2%)
Pupillary block	4 (8%)
Persistent epithelial defect	3 (6%)
Cystoid macular edema	1 (2%)
Graft rejection	1 (2%)

**Table 5:** Correlation between operative procedure and postoperative best spectacle-corrected visual acuity at 3 months.

	Postoperative visual acuity at 3 months						
		>20/63	<20/63–20/200	<20/200–20/400	<20/400	Total	*P* value
Operative procedure	DMEK^*∗*^	12	18	2	1	33	0.522
	DMEK + phacoemulsification	6	4	0	0	10	

Total		18	22	2	1	43	

^*∗*^DMEK: Descemet membrane endothelial keratoplasty.

**Table 6:** Correlation between donor age and postoperative best spectacle-corrected visual acuity at 3 months.

	Postoperative visual acuity at 3 months						
		>20/63	<20/63–20/200	<20/200–20/400	<20/400	Total	*P* value
Donor age	<50 years	2	11	0	0	13	0.03
	>50 years	16	11	2	1	30	

Total		18	22	2	1	43	

**Table 7:** Correlation between indication for surgery and postoperative best spectacle-corrected visual acuity at 3 months.

	Postoperative visual acuity at 3 months						
		>20/63	<20/63–20/200	<20/200–20/400	<20/400	Total	*P* value
Diagnosis	Fuchs	9	5	2	0	16	0.268
	PBK^*∗*^	6	16	0	1	23	
	Failed graft	2	1	0	0	3	
	Others	1	0	0	0	1	

Total		18	22	2	1	43	

^*∗*^PBK: pseudophakic bullous keratopathy.

## Data Availability

The data used to support the findings of this study are included within the supplementary information file.

## Supplementary Materials

Supplementary Materials The data used to support the findings of this study are included within the supplementary information file.

## References

[1] Melles G. R. J., Lander F., Rietveld F. J. R. (2002). Transplantation of descemet’s membrane carrying viable endothelium through a small scleral incision. *Cornea*.

[2] Melles G. R. (2006). *Posterior Lamellar Keratoplasty: DLEK to DSEK to DMEK*.

[3] Melles G. R. J., Ong T. S., Ververs B., van der Wees J. (2006). Descemet membrane endothelial keratoplasty (DMEK). *Cornea*.

[4] Price M. O., Price F. W. (2010). Descemet membrane endothelial keratoplasty. *International Ophthalmology Clinics*.

[5] Guerra F. P., Anshu A., Price M. O., Giebel A. W., Price F. W. (2011). Descemet’s membrane endothelial keratoplasty: prospective study of 1-year visual outcomes, graft survival, and endothelial cell loss. *Ophthalmology*.

[6] Price M. O., Giebel A. W., Fairchild K. M., Price F. W. (2009). Descemet’s membrane endothelial keratoplasty: prospective multicenter study of visual and refractive outcomes and endothelial survival. *Ophthalmology*.

[7] Dapena I., Ham L., Droutsas K., van Dijk K., Moutsouris K., Melles G. R. J. (2011). Learning curve in descemet’s membrane endothelial keratoplasty: first series of 135 consecutive cases. *Ophthalmology*.

[8] Dapena I., Yeh R. Y., Baydoun L. (2013). Potential causes of incomplete visual rehabilitation at 6 Months postoperative after descemet membrane endothelial keratoplasty. *American Journal of Ophthalmology*.

[9] Rodríguez-Calvo-de-Mora M., Quilendrino R., Ham L. (2015). Clinical outcome of 500 consecutive cases undergoing descemet’s membrane endothelial keratoplasty. *Ophthalmology*.

[10] Peraza-Nieves J., Baydoun L., Dapena I. (2017). Two-year clinical outcome of 500 consecutive cases undergoing descemet membrane endothelial keratoplasty. *Cornea*.

[11] Ham L., Dapena I., van Luijk C., van der Wees J., Melles G. R. J. (2009). Descemet membrane endothelial keratoplasty (DMEK) for Fuchs endothelial dystrophy: review of the first 50 consecutive cases. *Eye*.

[12] Tourtas T., Laaser K., Bachmann B. O., Cursiefen C., Kruse F. E. (2012). Descemet membrane endothelial keratoplasty versus descemet stripping automated endothelial keratoplasty. *American Journal of Ophthalmology*.

[13] Eye O. A. S. (2011). Standardized “no-touch” technique for descemet membrane endothelial keratoplasty. *Archives of Ophthalmology*.

[14] Groeneveld-van Beek E. A., Lie J. T., van der Wees J., Bruinsma M., Melles G. R. J. (2013). Standardized “no-touch” donor tissue preparation for DALK and DMEK: harvesting undamaged anterior and posterior transplants from the same donor cornea. *Acta Ophthalmologica*.

[15] Deng S. X., Lee W. B., Hammersmith K. M. (2018). Descemet membrane endothelial keratoplasty: safety and outcomes. *Ophthalmology*.

[16] Singh A., Zarei-Ghanavati M., Avadhanam V., Liu C. (2017). Systematic review and meta-analysis of clinical outcomes of descemet membrane endothelial keratoplasty versus descemet stripping endothelial keratoplasty/descemet stripping automated endothelial keratoplasty. *Cornea*.

[17] Schrittenlocher S., Schaub F., Hos D., Siebelmann S., Cursiefen C., Bachmann B. (2018). Evolution of consecutive descemet membrane endothelial keratoplasty outcomes throughout a 5-year period performed by two experienced surgeons. *American Journal of Ophthalmology*.

[18] Maier A. K. B., Gundlach E., Schroeter J. (2015). Influence of the difficulty of graft unfolding and attachment on the outcome in descemet membrane endothelial keratoplasty. *Graefe’s Archive for Clinical and Experimental Ophthalmology*.

[19] Schaub F., Enders P., Zachewicz J. (2016). Impact of donor age on descemet membrane endothelial keratoplasty outcome: evaluation of donors aged 17-55 years. *American Journal of Ophthalmology*.

[20] Dirisamer M., Ham L., Dapena I. (2011). Efficacy of descemet membrane endothelial keratoplasty: clinical outcome of 200 consecutive cases after a learning curve of 25 cases. *Archives of Ophthalmology*.

[21] Dirisamer M., van Dijk K., Dapena I. (2012). Prevention and management of graft detachment in descemet membrane endothelial keratoplasty. *Archives of Ophthalmology*.

[22] Liarakos V. S., Dapena I., Ham L., van Dijk K., Melles G. R. J. (2013). Intraocular graft unfolding techniques in descemet membrane endothelial keratoplasty. *JAMA Ophthalmology*.

[23] Flockerzi E., Maier P., Böhringer D. (2018). Trends in corneal transplantation from 2001 to 2016 in Germany: a report of the DOG-section cornea and its keratoplasty registry. *American Journal of Ophthalmology*.

[24] Chaurasia S., Price F. W., Gunderson L., Price M. O. (2014). Descemet’s membrane endothelial keratoplasty: clinical results of single versus triple procedures (combined with cataract surgery). *Ophthalmology*.

[25] Gogate P. (2009). Small incision cataract surgery: complications and mini-review. *Indian Journal of Ophthalmology*.

[26] Gogate P., Optom J., Deshpande S., Naidoo K. (2015). Meta-analysis to compare the safety and efficacy of manual small incision cataract surgery and phacoemulsification. *Middle East African Journal of Ophthalmology*.

[27] Singh S., Winter I., Surin L. (2009). Phacoemulsification versus small incision cataract surgery (SICS): which one is a better surgical option for immature cataract in developing countries?. *Nepalese Journal of Ophthalmology*.

[28] Benatti C. A., Tsao J. Z., Afshari N. A. (2017). Descemet membrane detachment during cataract surgery: etiology and management. *Current Opinion in Ophthalmology*.

[29] Lee W. B., Jacobs D. S., Musch D. C., Kaufman S. C., Reinhart W. J., Shtein R. M. (2009). Descemet’s stripping endothelial keratoplasty: safety and outcomes: a report by the American academy of ophthalmology. *Ophthalmology*.

[30] Hayashi T., Oyakawa I., Kato N. (2017). Techniques for learning descemet membrane endothelial keratoplasty for eyes of asian patients with shallow anterior chamber. *Cornea*.

[31] Veldman P. B., Dye P. K., Holiman J. D. (2016). The S-stamp in descemet membrane endothelial keratoplasty safely eliminates upside-down graft implantation. *Ophthalmology*.

[32] Jacob S., Agarwal A., Agarwal A., Narasimhan S., Kumar D. A., Sivagnanam S. (2014). Endoilluminator-assisted transcorneal illumination for descemet membrane endothelial keratoplasty: enhanced intraoperative visualization of the graft in corneal decompensation secondary to pseudophakic bullous keratopathy. *Journal of Cataract and Refractive Surgery*.

[33] Leon P., Parekh M., Nahum Y. (2018). Factors associated with early graft detachment in primary descemet membrane endothelial keratoplasty. *American Journal of Ophthalmology*.

[34] Heinzelmann S., Hüther S., Böhringer D., Eberwein P., Reinhard T., Maier P. (2014). Influence of donor characteristics on descemet membrane endothelial keratoplasty. *Cornea*.

[35] de Mora M. R. C., Groeneveld-van Beek E. A., Frank L. E. (2016). Association between graft storage time and donor age with endothelial cell density and graft adherence after descemet membrane endothelial keratoplasty. *JAMA Ophthalmology*.

